# Cord around the neck syndrome

**DOI:** 10.1186/1471-2393-12-S1-A6

**Published:** 2012-08-28

**Authors:** Morarji Peesay

**Affiliations:** 1 Montgomery General Hospital/Georgetown University Hospital, Washington DC, USA

## 

A nuchal cord (or Cord-Around-the Neck (CAN)) occurs when the umbilical cord becomes wrapped around the fetal neck 360 degrees. Nuchal cords are very common, the incidence of nuchal cord increases with advancing gestation from 12% at 24 to 26 weeks to 37% at term [1]. Most are not associated with perinatal morbidity and mortality. In some fetuses and newborns CAN may cause problems, especially when the cord is tightly wrapped around the neck. The cluster of cardiorespiratory and neurological signs and symptoms associated with unique physical features that occur secondary to tight cord-round-the-neck has been referred to as 'tCAN syndrome' (tight Cord Around the Neck Syndrome) [2]. A small number of studies have shown that nuchal cord and or tCAN can affect the outcome of delivery and may have long-term effects on the infant [3] and but as a causative factor for stillbirth it is debatable [4,5]. However, some case reports of postmortem findings on stillbirths show negative pathology reports and tight cord around the neck being the only cause of death [6].

It is the unique physical features of tCAN syndrome that distinguishes it from birth asphyxia even though there are many similarities between these two conditions. Umbilical cord abnormalities are considered as one of the causative factor for birth asphyxia. The manifestation of tCAN symptomatology seems to happen both in the presence of normal and depressed AGPAR scores [7]. Umbilical cord compression due to tCAN may cause obstruction of blood flow first in thin walled umbilical vein, while infant’s blood continues to be pumped out of baby through the thicker walled umbilical arteries thus causing hypovolemia and hypotension resulting in acidosis [8]. Anemia [9] and mild respiratory distress may occur. Some of these infants may also have facial and conjuctival petechiae [10] and rarely petechiae of the neck and upper part of the chest and skin abrasion of neck [11] where the cord was tightly wrapped and facial suffusion [12], all of which can also be seen in some postmortem findings of stillbirth infants who had tCAN [Archana Bargaje, personal communication]. If born alive, some of these infants may also be somewhat obtunded with a low tone and have transient feeding difficulties. These findings raise the possibility of transient encephalopathy, which may lead to long-term complications.

A stillbirth attributed to a cord problem should have evidence of cord obstruction or circulatory compromise. Other potential causes of stillbirth need to be excluded prior to labelling cord abnormalities as the causative factor, since cord abnormalities seen in more than a third of all normal live births.

The tCAN Syndrome may conceptually be similar to strangulation which may result in non lethal problems or death. The pathophysiological mechanisms of strangulation injuries (lethal and non lethal) involves venous, arterial obstruction (arterial spasm due to carotid pressure) in the neck and vagal collapse (increased parasympathetic tone) [13]. This can lead to cerebral stagnation, hypoxia, and unconsciousness, which, in turn, produces loss of muscle tone. The same pathophysiology of strangulation may possibly be applicable to tCAN syndrome in neonates. A study on potentially asphyxiating conditions and spastic cerebral palsy in infants of normal birth weight showed evidence of association of tCAN in children with quadriplegia [14].

Intermittent umbilical cord occlusion in preterm and near term sheep caused a decline in pO_2_ and pH, and higher PCO_2_ and altered brain protein synthesis/degradation [6]. Whether human fetal intermittent strangulation by tCAN have similar brain protein alterations and thus long-term effects remains to be seen. Using specific placental histologic criteria for umbilical blood flow restriction in unexplained stillbirth Parast et al [4] showed significant correlation of placental changes of “minimal histologic criteria” with cord accidents (as tCAN is part of cord accidents). Nuchal cords showed highest rates of thrombosis-related placental histopathology and fetal thrombotic vasculopathy and thrombosis seems to be highly specific for cord related stillbirths [4,5].
